# Factors related to the waiting time for scheduling an oral biopsy in Brazil: a multilevel analysis

**DOI:** 10.1186/s12913-023-09437-0

**Published:** 2023-05-09

**Authors:** Alessandro Diogo De-Carli, Amanda Ramos da Cunha, Gleyson Kleber do Amaral-Silva, Jader Vasconcelos, Mara Lisiane de Moraes dos Santos, Livia Fernandes Probst

**Affiliations:** 1 Faculty of Dentistry, Federal University of Mato Grosso do Sul, Campo Grande, Brazil; 2 School of Public Health, University of São Paulo, São Paulo, Brazil; 3 Municipal Health Secretariat, Campo Grande, Brazil; 4 Integrated Health Institute, Federal University of Mato Grosso do Sul, Campo Grande, Brazil; 5 Health Technology Assessment Unit, Oswaldo Cruz German Hospital, São Paulo, Brazil

**Keywords:** Public Health Dentistry, Mouth Neoplasms, Secondary care, Public Health Systems Research, Community Dentistry, Biopsy, Appointments and schedules

## Abstract

**Background:**

Timely diagnosis of oral cancers is critical, and performing biopsies of oral lesions with suspected malignancy is a crucial step in achieving this goal. The waiting time for the diagnosis may be related to the progression and prognosis of malignant neoplasms.

**Objective:**

The aim of this observational, cross-sectional, national-level study was to identify the factors associated with the waiting time for scheduling an oral biopsy, based on the identification of its need.

**Methods:**

We used secondary data from the Brazilian public health system, obtained from the 2nd cycle of the National Program to Improve Access and Quality of Dental Specialty Centers (PMAQ-CEO). The study outcome was the waiting time for scheduling an oral biopsy, starting from the identification of the need for the exam. We analyzed individual and contextual variables using multilevel statistical analysis.

**Results:**

In 51.8% of DSC the waiting time for scheduling a biopsy was non-immediate; in 58.1% of CEOs, the sum of the weekly workload of dentists working in the Stomatology specialty is up to 20 h per week; in terms of coverage, 67.1% of the CEOs have only municipal coverage and 34.0% are references for up to 12 oral health teams in primary health care; only the coverage variable remained significant in the multivariate model (p < 0.05). Of the contextual variables, none of the variables remained significant (p > 0.05). When these were analyzed together, only the coverage remained significant (p < 0.05);

**Conclusion:**

Our analysis indicates that the waiting time for scheduling an oral biopsy is longer in CEOs that cover only one municipality and is not related to contextual factors.

## Background

Oral cancer represents an important public health problem in Brazil [1] and worldwide [2, 3], especially in developing countries. According to GLOBOCAN estimates, supplied by the International Agency for Research on Cancer, about 377,000 new cases and 177,000 deaths were caused by oral cancer in the world in 2020 [4]. For that same year, the estimated incidence and mortality rates for Brazil for this disease were, respectively, the highest and second highest in South America. Oral cancer is the fifth most incident type of neoplasm among men in Brazil and has maintained this position for many years [5].

The chance of survival from oral cancer – whose average estimate is 50% in 5 years [3, 6, 7] – is associated with tumor staging at the time of the diagnosis and the start of treatment [6, 8, 9]. The availability of access to health services that offer the necessary care in a timely manner is crucial for this outcome. The delay in diagnosing this disease is a matter of concern and a prevalent one – most oral neoplasms are detected at an advanced stage [10–12]. A study that analyzed the relative survival of oral cancer in several health institutions in South America identified that around 48% of the patients included in the study were diagnosed with tumors at the most advanced stage, i.e., stage IV. The mortality among these patients was dramatically higher (about eleven-fold) than among those diagnosed with early-stage lesions [6].

The Unified Health System (SUS, *Sistema Único de Saúde*) is the Brazilian public health system that provides care through multiprofessional teams, including Community Health Agents (CHAs), delivering services to a specific population within a designated territory. In accordance with the National Oral Health Policy (PNSB, *Política Nacional de Saúde Bucal*), SUS also offers comprehensive oral health care to the Brazilian population [13, 14]. Therefore, in this system, oral cancers should be preferably investigated at Dental Specialty Centers (CEOs, *Centros de Especialidades Odontológicas*). These comprise the Secondary Oral Health Care (ASSB, *Atenção Secundária em Saúde Bucal*), being a reference for the Oral Health teams (eSB, *equipes de Saúde Bucal*) of Primary Health Care (PHC), in Brazil mainly represented by the Family Health Strategy (ESF, *Estratégia Saúde da Família*). Faced with the diagnosis of malignancy or in the presence of lesions that require a complex surgical-ambulatory approach in the head and neck region, a referral is made to Tertiary Health Care (THC) for treatment and rehabilitation at hospital level [13]. This constitutes the Oral Health Care Network (RASSB, *Rede de Atenção à Saúde Bucal* ) is constituted in Brazil.

The Brazilian ASSB can be considered as having been recently implemented, as it has only been consolidated since 2004, with the implementation of the PNSB. Therefore, despite significant improvements in oral health care at all levels of health care in the SUS [15–17], the ASSB still has weaknesses in terms of supply and access to services, including those related to Stomatology. This specialty, which should be offered in all CEOs, has the responsibility, among others, of diagnosing and referring cases of oral cancer [17, 18] to the THC. Previous studies highlighted, at a national level, important weaknesses in relation to coverage, access, work process, infrastructure and performance of specific Stomatology procedures, mainly biopsies [17, 18], the gold standard clinical procedure for diagnosis and/or treatment of several oral lesions, including oral cancer – whose diagnosis is made through an incisional biopsy followed by histological evaluation [19].

Assuming that the waiting time for the diagnosis may be related to disease progression in the case of malignant neoplasms and, consequently, to the prognosis of these cases, the interval between the moment when the need for the biopsy is identified and scheduling the biopsy is an outcome of the care network related to this disease that deserves attention. Although the biopsy is not the only procedure performed in lesions suspected of malignancy, these necessarily require this procedure – which, within the scope of the SUS, is preferably performed in the CEOs.

From this perspective, identifying the factors that are involved in this waiting time can contribute to the detection of weak points in the care network and the optimization of flows, which is necessary considering the high rates of late oral cancer diagnosis in the country. Therefore, the aim of this study was to analyze, at a national level, which factors are associated with the waiting time for scheduling an oral biopsy at the CEO, starting with the identification of the need for the exam.

## Method

### Ethical aspects

The microdata used in this study were obtained from national information systems with public and unrestricted access. Therefore, it was not necessary to obtain approval from to the Research Ethics Committee.

### Study design and context

This was an analytical, cross-sectional, epidemiological, national-base study that used secondary data and was reported in accordance with the *STrengthening the Reporting of OBservational studies in Epidemiology* (STROBE) guidelines [20]. This study uses data from the external evaluation of the 2nd cycle of the National Program to Improve Access and Quality of Dental Specialty Centers (PMAQ-CEO, *Programa Nacional de Melhoria do Acesso e da Qualidade dos Centro de Especialidades Odontológicas*), carried out in 2018, in Brazil [21].

The PMAQ-CEO was established within the scope of the National Oral Health Policy through Ordinance Number 261/GM/MS, of February 21, 2013, and its main objective was to increase access and improve the quality of CEOs throughout Brazil. Microdata related to module II, which evaluated the work process of the CEOs, were used.

### Study universe and sample

The study universe comprised the Dental Specialty Centers that joined the program and answered the external evaluation questionnaire and their respective municipalities. Of the 1097 CEOs operating in Brazil in 2018, 1042 answered the external evaluation questionnaire. Of these, 142 were excluded for not answering the outcome question. Therefore, the final sample comprised 900 CEOs from 745 municipalities in Brazil, which correspond to 82.04% of the total establishments operating in the Brazilian territory in 2018.

### Analyzed variables

**Fig. 1:**
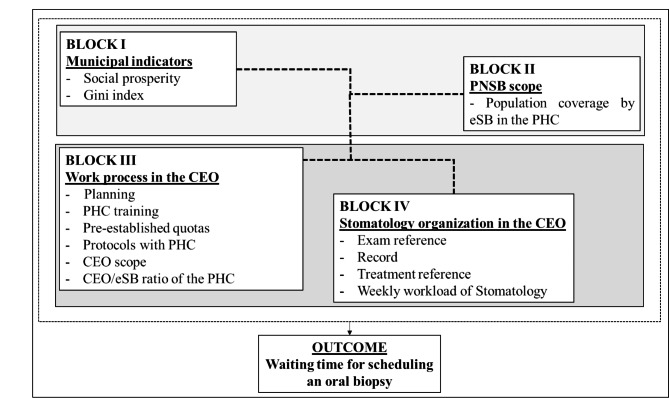
Theoretical model adapted for the study

The outcome established in this study was the waiting time for scheduling an oral biopsy, starting from the identification of the need for the exam. The independent variables were organized into four blocks, according to the adopted theoretical model [22], as shown in Fig. 1. The waiting time for scheduling a biopsy was dichotomized into “Up to one day” and “More than one day up to 60 days”, since it was assumed that, if carried out in up to one day, the scheduling would be characterized as immediate, that is, without waiting time, which would represent the ideal scenario in this stage of the care chain [21].

The variables used in the study, the way they were treated and the sources from which they were collected are shown in Chart 1. For the analyses, the independent variables were divided into individual (related to the CEO; blocks III and IV) and contextual (related to the municipalities; blocks I and II).

**Chart 1:** Variables (outcome and independent) used in the study

Variable	Definition	
***Outcome***		
Waiting time for scheduling a biopsy, starting from the identification of the need for the exam*	Up to one day	
	More than one day up to 60 days	
***Block IV individual independent variables (related to the CEO): stomatology organization***		
It has a reference where the anatomohistopathological exam will be sent *	Yes	
	No	
There is a record of users with suspected/diagnosed oral cancer*	Yes	
	No	
It has a reference where the confirmed cases of oral cancer will be sent *	Yes	
	No	
Sum of the weekly workload of dentists working in Stomatology *	Number	
***Block III individual independent variables (related to the CEO): CEO work process***		
Action planning and regular evaluations*	Yes	
	No	
Training with PHC professionals for the detection of oral cancer*	Yes	
	No	
Predefined quotas by PHC eSB for referral to Stomatology *	Yes	
	No	
	Does not have this specialty	
Clinical protocols for referral of patients from PHC to the CEO to Stomatology *	Yes	
	No	
	Does not have this specialty	
Scope of the CEO only for this municipality *	Yes	
	No	
This CEO is a reference for how many eSB of PHC *	Number	
***Block II contextual independent variables (related to the municipalities): municipal organization of oral health***		
Oral health coverage ^#^	Variable that represents the proportion of the population covered by oral health at the PHC level, calculated using the formula: (n. of eSFSB * 3450) + (n. of eASB param + n. of equivalent eSFSB)*3000 *100 Population estimate For the analysis, this variable was dichotomized: ≤ 64.39% (median) > 64.39%	
***Block I contextual independent variables (related to the municipalities): municipal indicators***		
Social Prosperity ^&^	It represents the simultaneous occurrence of high human development with low social vulnerability. It is divided into the following categories: Very High, High, Medium, Low and Very Low.	
Gini index^&^	A value equal to zero represents the situation of total equality, that is, everyone has the same income. For the analysis, this variable was dichotomized: ≤ 0.52 (median) > 0.52	

**Data source** * National Program to Improve Access and Quality of Dental Specialty Centers (PMAQ-CEO)

^#^ Health Information System for Primary Care (SISAB, *Sistema de Informação em Saúde para a Atenção Básica*)

^&^*Instituto de Pesquisa Econômica Aplicada* (IPEA) [23]

### Data organization and statistical analysis

All secondary data were organized in spreadsheets that comprised the researcher’s own database, using the Microsoft Excel program.

Descriptive analyses of variables were performed using absolute and relative frequencies, followed by crude analyses of each variable in relation to the outcome. For this purpose, simple logistic regression models were used. The degree of association between the variables was described by crude odds ratios with the respective 95% confidence intervals. Then, a multilevel multiple regression was performed, considering the variables that had p < 0.20 in the crude analyses. In the first step, the contextual effect was evaluated from an empty model, using the intraclass correlation coefficient to verify the part of the total variation that was due to the municipal variables. Subsequently, three models were tested, only with individual level variables (CEOs), only with contextual level variables (municipalities) and with both individual and contextual levels. In all models, the CEOs were considered nested within the municipalities, since more than one CEO per municipality was evaluated. Based on multiple multilevel models, the adjusted odds ratios were estimated, with their respective 95% confidence intervals. The fit of the models was evaluated using the QICc (Quasi-likelihood information criterion). The analyses were performed using the R program.

## Results

Data from 900 CEOs from 745 Brazilian municipalities were evaluated. According to the data in Table 1, in 51.8% of the CEOs, the waiting time for scheduling a biopsy, starting from the identification of the need for the exam, was not immediate. It was observed that 95.8% of the CEOs have references where the biopsy specimens are sent for anatomohistopathological examination; 73.4% have records of users with suspected or diagnosed oral cancer and 94.0% have references for confirmed cases of oral cancer. In 58.1% of CEOs, the sum of the weekly workload of dentists working in the Stomatology specialty is up to 20 h. The actions developed in 74.2% of the CEOs originate from planning and regular evaluations and in 65.0% of them the professionals carry out the training with primary care professionals for the detection of oral cancer. In 75.8% there are predefined quotas established by the oral health teams of Primary Health Care for referring users to Stomatology and in 68.8% there are agreed clinical protocols that guide the referral of patients from primary health care to the CEO to Stomatology. As for coverage, 67.1% of the CEOs are municipal only and 34.0% are references for up to 12 primary care oral health teams.

**Table 1:** Crude analyses of associations with the deadline for the scheduling of a biopsy at Dental Specialty Centers, starting from the identification of the need for the exam

Variable		Category	n (%)	Deadline		Crude OR (95%CI)	p-value
				Up to one day	*More than one day		
				n (%)	n (%)		
**General sample**			900 (100.0%)	434 (48.2%)	466 (51.8%)		
**CEO variables**							
	The CEO is a reference center to carry out the anatomohistopathological analysis of biopsy specimens	Yes	862 (95.8%)	418 (48.5%)	444 (51.5%)	Ref	
		No	38 (4.2%)	16 (42.1%)	22 (57.9%)	1.29 (0.67–2.50)	0.4418
	The CEO has a record of users with suspected or diagnosed oral cancer	Yes	661 (73.4%)	343 (51.9%)	318 (48.1%)	Ref	
		No	239 (26.6%)	91 (38.1%)	148 (61.9%)	1.75 (1.30–2.37)	0.0003
	The CEO is a reference center to receive confirmed cases of oral cancer	Yes	846 (94.0%)	410 (48.5%)	436 (51.5%)	Ref	
		No	54 (6.0%)	24 (44.4%)	30 (55.6%)	1.18 (0.68–2.04)	0.5670
	Sum of the weekly workload of dentists working in the minimum specialties (Stomatology)	≤ 20 h (median)	523 (58.1%)	246 (47.0%)	277 (53.0%)	1.35 (0.95–1.93)	0.0922
		> 20 h	163 (18.1%)	89 (54.6%)	74 (45.4%)	Ref	
		Not applicable	214 (23.8%)	99 (46.3%)	115 (53.7%)	-	
	Are the actions developed in this CEO the result of planning and periodic evaluations?	Yes	668 (74.2%)	325 (48.7%)	343 (51.3%)	Ref	
		No	232 (25.8%)	109 (47.0%)	123 (53.0%)	1.07 (0.79–1.44)	0.6610
	The CEO professionals carry out training with primary care professionals for the detection of oral cancer	Yes	585 (65.0%)	298 (50.9%)	287 (49.1%)	Ref	
		No	315 (35.0%)	136 (43.2%)	179 (56.8%)	1.37 (1.04–1.80)	0.0264
	There are quotas pre-defined by the Primary Care oral health team to refer users to stomatology	Yes	104 (11.6%)	51 (49.0%)	53 (51.0%)	Ref	
		No	682 (75.8%)	328 (48.1%)	354 (51.9%)	1.04 (0.69–1.57)	0.8575
		Does not have this specialty	114 (12.7%)	55 (48.2%)	59 (51.8%)	1.03 (0.61–1.76)	0.9069
	There are agreed clinical protocols that guide the reference of patients from primary care to the CEO to stomatology	Yes	619 (68.8%)	306 (49.4%)	313 (50.6%)	Ref	
		No	281 (31.2%)	128 (45.6%)	153 (54.4%)	1.17 (0.88–1.55)	0.2802
	This CEO has a municipal scope only (it is a reference only for this municipality)	Yes	604 (67.1%)	277 (45.9%)	327 (54.1%)	1.33 (1.01–1.76)	0.0431
		No	296 (32.9%)	157 (53.0%)	139 (47.0%)	Ref	
	This CEO is a reference for how many primary care oral health teams?	≤ 12 (median)	306 (34.0%)	140 (45.8%)	166 (54.2%)	1.01 (0.73–1.39)	0.9564
		> 12	298 (33.1%)	137 (46.0%)	161 (54.0%)	Ref	
		Not applicable	296 (32.9%)	157 (53.0%)	139 (47.0%)	-	
**Municipality variables**							
	Oral health coverage	≤ 64.39% (median)	450 (50.0%)	228 (50.7%)	222 (49.3%)	Ref	
		> 64.39%	450 (50.0%)	206 (45.8%)	244 (54.2%)	1.22 (0.94–1.58)	0.1424
	Social Prosperity	Very high	456 (50.7%)	228 (50.0%)	228 (50.0%)	Ref	
		High	106 (11.8%)	49 (46.2%)	57 (53.8%)	1.16 (0.76–1.78)	0.4842
		Medium	125 (13.9%)	58 (46.4%)	67 (53.6%)	1.15 (0.78–1.72)	0.4759
		Low	105 (11.7%)	55 (52.4%)	50 (47.6%)	0.90 (0.59–1.39)	0.6600
		Very low	108 (12.0%)	44 (40.7%)	64 (59.3%)	1.45 (0.95–2.22)	0.0843
	Gini Index	≤ 0.52 (median)	494 (54.9%)	246 (49.8%)	248 (50.2%)	Ref	
		> 0.52	406 (45.1%)	188 (46.3%)	218 (53.7%)	1.15 (0.88–1.50)	0.2969

*Outcome event. Ref: Reference category for independent variables. OR: Odds ratio. CI: Confidence interval

Table 2 depicts the results of the multiple multilevel logistic regression analysis, starting from the variables that showed p < 0.20 in the crude analyses. The intraclass correlation coefficient was 0.2189, indicating that 21.9% of the total variation is due to municipality variables. In model 1 (individual variables), only the variable coverage remained significant in the multivariate model (p < 0.05). In model 2, of the contextual variables, none of the variables remained significant (p > 0.05). When these variables were analyzed together (model 3) only coverage remained significant (p < 0.05). As the Quasi-Likelihood Information Criterion (QICc) of the model with only CEO variables was lower and the lower the value of the QICc, the better the model, the final model comprised only the CEO variables. Thus, CEOs with municipal coverage only are more likely to have a waiting time for scheduling a biopsy longer than one day (OR = 1.46; 95%CI: 1.05–2.03), p < 0.05. Among the CEOs with municipal coverage only, 54.1% have a waiting time for scheduling a biopsy of more than one day, while among those which are also a reference for other municipalities, this percentage is 47.0%.

**Table 2:** Multilevel analysis of associations with the deadline for scheduling a biopsy at Dental Specialty Centers, starting from the identification of the need for the exam

Variable		Category	Model 1 (Individual variables)		Model 2 (contextual variables)		Model 3 (individual and contextual variables)		Final model	
			Adjusted OR (95%CI)	p-value	Adjusted OR (95%CI)	p-value	OR (final model)	p-value	OR (final model)	p-value
**CEO variables**										
	The CEO has a record of users with suspected or diagnosed oral cancer	Yes	Ref				Ref		Ref	
		No	1.37 (0.92–2.06)	0.1227			1.36 (0.90–2.05)	0.1447	1.37 (0.92–2.06)	0.1227
	Sum of the weekly workload of dentists working in the minimum specialties (Stomatology)	≤ 20 h (median)	1.34 (0.93–1.92)	0.1149			1.30 (0.90–1.87)	0.1599	1.34 (0.93–1.92)	0.1149
		> 20 h	Ref				Ref		Ref	
		Not applicable	-				-		-	
	The CEO professionals carry out training with primary care professionals for the detection of oral cancer	Yes	Ref				Ref		Ref	
		No	1.31 (0.94–1.81)	0.1054			1.32 (0.95–1.83)	0.0948	1.31 (0.94–1.81)	0.1054
	This CEO has a municipal scope only (it is a reference only for this municipality)	Yes	1.46 (1.05–2.03)	0.0229			1.46 (1.04–2.04)	0.0287	1.46 (1.05–2.03)	0.0229
		No	Ref				Ref		Ref	
**Municipality variables**										
	Oral health coverage	≤ 64.39% (median)	-	-	Ref		Ref		-	-
		> 64.39%			1.21 (0.89–1.66)	0.2211	1.29 (0.90–1.85)	0.1687	-	-
	Social Prosperity	Very high	-	-	Ref		Ref			
		High			1.07 (0.68–1.68)	0.7717	1.03 (0.61–1.75)	0.9088	-	-
		Median			1.06 (0.69–1.63)	0.7878	1.07 (0.66–1.73)	0.7923	-	-
		Low			0.83 (0.53–1.30)	0.4148	0.74 (0.44–1.27)	0.2785	-	-
		Very low			1.29 (0.81–2.05)	0.2842	1.32 (0.73–2.39)	0.3510	-	-
QICc (Quasi-likelihood information criterion)			943.22		1.253.38		947.36		943.22	

Ref: Reference category for independent variables. OR: Odds ratio. CI: Confidence interval. Variance between municipalities = 0.2196; Residual variance = 0.7834; ICC: Intraclass correlation coefficient (Part of the total variation that is due to the contextual level - Municipalities) = 0.2189 QIC (empty model) = 1,248.53

## Discussion

The results of this study indicate that, for a significant part of the investigated CEO, when the need for oral biopsies is identified, these are not scheduled immediately. This is a result that deserves to be registered, considering that the performance of the biopsy is a critical step for the diagnosis of lesions suspected of malignancy. This finding is aggravated by the fact that, between the first and second cycles of the PMAQ-CEO, there was a decrease in the number of establishments that performed biopsies [18].

Once the malignancy is confirmed, the SUS user still faces a series of limitations in terms of access to cancer treatment at the THC level, although there are national laws that ensure a period of up to 30 days for carrying out the necessary tests to confirm cases when the main diagnostic hypothesis is malignant neoplasm [24]; and, in case of confirmed malignancy, the SUS user has the right to start their treatment within 60 days after the histopathological report is rendered [25]. Thus, the non-immediate scheduling of the biopsy may be an additional obstacle to detecting and treating oral cancer in a timely manner.

From this perspective, it should be noted that, in more than 60% of the CEO, the histopathological report is available 15 days after the biopsy is performed; however, this period can be as long as two months [17]. An extended wait is a matter of concern particularly in a country like Brazil, which has rising mortality rates from oral cancer in most regions and where, in about 74% of cases, it takes the patient more than 60 days to start the treatment [1]. Moreover, in Brazil [10, 16] and worldwide [11, 12], oral cancer is still predominantly diagnosed at an advanced stage.

The relationship between PHC and ASSB is important to promote health care. The greater number of appointments in the PHC until the decision for referral to the ASSB and the degree of refinement of protocols, the worse the prognosis and outcomes for confirmed cases of oral and oropharyngeal cancer [11, 12]. In this sense, training with PHC professionals for the detection of oral cancer is considered a relevant strategic standard of the work process, comprising a CEO evaluation indicator [14]. The fact that 35% of CEO professionals do not comply with this guideline may explain delays in referring the user (extension of PHC care time in the pre-reference period) and the increase in undue references. Moreover, it should be noted that in Brazil, other weaknesses also occur at the THC level that can aggravate this situation, considering that between the first and second cycles of the PMAQ-CEO, there was a decrease of 18.3% of the number of CEOs that had reference hospitals for confirmed cases of oral cancer [18], where the oral cancer cases are treated.

Specific aspects of human resources in the area of Stomatology, the specialty responsible for investigating these cases, may aggravate this situation, considering that in most CEOs investigated in this study, the workload for the specialty was only 20 h per week. In addition, it is plausible to consider that matters related to the infrastructure and availability of materials directly related to the feasibility of performing biopsies in the CEOS [17] interfere with the delay in receiving the histopathological report, and, in case of malignancy, consequently, it increases the time to start the appropriate treatment. As a relevant factor, we emphasize that the performance of biopsies is not included in the Stomatology goals in CEO, which can lead to the prioritization of other procedures by the management [26].

Although there have been improvements in relation to the registration of users with suspected/confirmed diagnosis of oral cancer between the first and second cycles of the PMAQ-CEO [18], in 26.6% of the CEOs this registration/control does not exist. This may reflect gaps in meeting the characteristic of longitudinality of care within the scope of PHC, the level of care responsible for monitoring cancer cases in the community. This improvement was also observed in relation to the implementation of clinical protocols for the referral of patients with suspected oral cancer to the Stomatology specialty [18]. However, in 31.2% of CEO, the lack of this clinical protocol persists, signaling a relevant limitation that can impair the coordination of care. These results reveal the importance, at the level of the work process, of promoting the dialogue between the PHC and the ASSB, considering that both characteristics, when absent or present to a low extent, imply a barrier to access oral health services, resulting in a significant repressed demand and long waiting periods for appointments and exams, distancing oral health care from the assumptions of health care networks [27].

Most CEO were a reference service for only one municipality, which did not guarantee the immediate scheduling of the biopsy exam, since in these establishments, there are 1.46-fold more chances of this scheduling being carried out within a period longer than one day. The CEO that are references for more than one municipality are faster in terms of scheduling the biopsy. In these places, procedural and organizational issues of the RASB, with better attainment of the previously mentioned characteristics, may be related to the better performance of these establishments in relation to the outcome variable.

Pre-defined quotas for the PHC eSB to refer users to Stomatology occur in most CEO. Although this suggests the organization of flows and the work process for the reference to the ASSB, it is worth reflecting that, despite the work overload and high demands, there may be stagnation of patients with urgent diagnostic needs in the PHC. In this sense, it is necessary to review strict protocols, considering the community orientation and the sociodemographic characteristics of the territory, so that the diagnosis can be attained within a shorter period of time. Despite the convergence between the PNSB and the National Policy for Cancer Prevention and Control, with the expansion of services in the last 15 years, population coverage remains low. This implies barriers to timely diagnosis and treatment, interfering with the quality of life and survival of users and increased costs for SUS, factors that could be mitigated through the implementation of processes of regionalization and universalization of access [26].

The faster scheduling of biopsies was not related to the contextual variables used in this study. Consequently, it is suggested that this outcome may be related to factors that are proximal to the work process, human, financial and infrastructure resources, or to more specific contextual determinants of the territories in which they are located.

This study has limitations inherent to the use of secondary data. Secondary data obtained from national information systems with public and unrestricted access offer numerous advantages, such as broad population coverage and low cost for collecting information. However, as these data are usually collected in routine health services without previous research purposes, the absence of important information for the analyses of interest can represent significant disadvantages [28].

In turn, the quality of information from databases can be assessed in two dimensions: completeness and accuracy. Completeness refers to the extent to which data are missing from the perspective of the outlined research question. Missing data are unavoidable; however, it is often necessary to understand the extent to which important variables are missing and the possible reasons for their absence. Another important dimension is accuracy. Information from electronic system records, such as procedure codes or numeric values, can sometimes be inaccurately recorded [28]. It is noteworthy that to ensure result reliability, transparent sources of information were used, approved by the Brazilian Ministry of Health. It is also noteworthy that the outcome does not refer exclusively to the scheduling of biopsies for suspected malignant lesions; this procedure is indicated for a wide variety of clinical conditions, which numerically surpass malignant lesions [29]. Therefore, the waiting time investigated in this study does not refer solely to that experienced by patients with oral cancer – but also experienced by those with other conditions.

As future investigation perspectives, our results highlight the urgent need to conduct in-depth studies that can contribute to expanding our knowledge about the critical aspects related to reference and counter-reference procedures in oral cancer care. These studies should consider the overall structure of the RASSB, the experiences of healthcare professionals and patients, and the connections between tertiary care and diagnostic laboratories. Furthermore, we must assess the average waiting time required to obtain the final microscopic diagnosis of diagnosed cancer cases to identify areas of improvement.

Another crucial aspect to consider in future research is the training and qualification of Dental Specialties Centers to diagnose oral lesions with suspected malignancy. Given the complexity of this issue, we recommend conducting mixed studies to identify and understand the specificities of care in this context, ultimately leading to implementation studies. By adopting a multidisciplinary approach and examining various factors, we can enhance our understanding of the challenges and opportunities for improving oral cancer care. This will pave the way for developing effective interventions that can benefit both healthcare professionals and patients.

The present study provided information on relevant aspects of care for oral cancer in the CEO in Brazil, reinforcing the need to consider it a priority in public oral health policies [30]. In the specific case of scheduling a biopsy, the reference time interval for the ASSB [12] may represent a strategic target for intervention, and its optimization could contribute to the meeting of time limits established by the Brazilian legislation for both the diagnosis and start of the cancer treatment. Therefore, protocols are required to ensure epidemiological surveillance and immediate referral of patients with lesions suspected of oral cancer, considering the occurrence of potentially severe sequelae in relation to prognosis, in the presence of delays between the identification of the lesion and the specialists’ opinion [27].

## Conclusion

It was concluded that the waiting time until the scheduling of biopsies is longer in Dental Specialties Centers with municipal coverage only (individual factor referring to the CEO’s work process). And we did not identify any significant contextual factors that influenced the waiting time for scheduling biopsies.

## Data Availability

Public data from National Program to Improve Access and Quality of Dental Specialty Centers (PMAQ-CEO) is available at https://aps.saude.gov.br/ape/pmaq/ciclo2ceo/. Public data from Health Information System for Primary Care (SISAB, *Sistema de Informação em Saúde para a Atenção Básica*) is available at https://egestorab.saude.gov.br/paginas/acessoPublico/relatorios/relHistoricoCobertura.xhtml. Public data from *Instituto de Pesquisa Econômica Aplicada* (IPEA) is available at http://www.ipeadata.gov.br/Default.aspx. All secondary data were organized in spreadsheets that comprised the researcher’s own database, using the Microsoft Excel program. The researcher’s own database used and analyzed during the current study is available from the corresponding author upon reasonable request.

## Abbreviations

ASSB: Secondary Oral Health Care (*Atenção Secundária em Saúde Bucal*)
CEO: Dental Specialties Center (*Centro de Especialidades Odontológicas*)
eSB: Oral Health Team (*Equipe de Saúde Bucal*)
ESF: Family Health Strategy (*Estratégia Saúde da Família*)
PHC: Primary Health Care
PMAQ-CEO: National Program to Improve Access to and Quality of Dental Specialty Centers (*Programa Nacional de Melhoria do Acesso e da Qualidade dos Centro de Especialidades Odontológicas*)
PNSB: National Oral Health Policy (*Política Nacional de Saúde Bucal*)
RASB: Oral Health Care Network (*Rede de Atenção à Saúde Bucal*)
SUS: Unified Health System (*Sistema Único de Saúde*)
THC: Tertiary Health Care
